# Phytochemistry in the Ethnopharmacology of North and Central America

**DOI:** 10.3389/fphar.2022.815742

**Published:** 2022-02-28

**Authors:** John Thor Arnason, Cory S. Harris, José A. Guerrero-Analco

**Affiliations:** ^1^ Biology Department, University of Ottawa, Ottawa, ON, Canada; ^2^ Red de Estudios Moleculares Avanzados, Instituto de Ecología A.C., Xalapa, Mexico

**Keywords:** phytochemistry, medicinal plants, chemometrics, activity biomarkers, dereplication, untargeted metabolomics

## Abstract

Traditionally the role of phytochemistry in the ethnopharmacology of North and Central America has been to characterize plant materials so that they can be produced reproducibly for commercial use or to identify active principles in unstudied traditional medicines for drug discovery. With new decolonial objectives coming from Indigenous communities, emphasis has shifted to evaluating the safety and efficacy of traditional medicines and preparations for community use. With new techniques and technologies available, scientific focus has shifted from individual bioactives to more rapid and comprehensive chemical characterizations and polypharmacy of traditional medicines. Untargeted metabolomics and associated statistical treatments have greatly expanded identification of components, improved species and cultivar identification and provided means for identifying multiple activity biomarkers, via chemometric and biochemometric analysis. New integrated techniques are available for identifying multiple active principles and synergists. The recent explosion of information is not without problems that need to be addressed including many unconfirmed tentative identifications of phytochemicals, lack of quantitative testing, superficial chemical activity testing and continuing need for dereplication.

## Introduction

There is no shortage of medicinal and other Indigenous uses for native plants in North America and Central America, known to Haudenosaunee and Anishinaabe indigenous peoples as Turtle Island, and the place of creation (Robinson, 2018). For example, Moerman et al., 2018) records 4029 taxa for ethnobotanically used plants in North America. For Central America, the Mesoamerican Medicinal Plant Database includes a total of 2188 plant taxa (Geck et al., 2020) but more regionally focussed reviews also reveal the traditional use of many species in some individual cultures, such as 350 by the Q’eqchi’ Maya (Arnason et al., 2022). Phytochemical and ethnopharmacological studies have only examined a small percentage of these plants and uses. Of these, the pharmacopeial or commercial medicinal plants are the best studied, but many Indigenous traditional medicines are now emerging as the focus of new research (Hall et al., 2022; Mata et al., 2022).

For commercial or pharmacopeial medicinal plants, characterization of the preparation is a fundamental step. Phytochemistry is essential to this so that any published experiment can be replicated or extended by others, and so that pharmacologically and clinically tested material can be shown to be consistent or comparable to preparations used in practice. Unlike single entity drugs, traditional medicines contain multiple active principles that have additive, antagonistic and synergistic effects, so that full characterization is of value but may present a difficult, expensive and time-consuming challenge for scientists. Many excellent ethnopharmacology studies have been rejected at publication because the authors could not provide adequate information on chemical characterization.

For the less well studied non-commercial Indigenous medicinal plants and herbal medicines of the Americas, the challenge is identifying bioactive molecules in the botanical drug, often with a goal of making new compound discoveries or for the purpose of validating and providing respect for traditional knowledge. Both the scientific and cultural context of this field has evolved rapidly in the last 15 years.

The goal of this insight review is to provide a discussion of issues and advances in phytochemistry of medicinal plants of the Americas taken from our experience as well as from selected examples of high impact peer reviewed papers in the literature. For example, an important area of discussion is decolonizing research in ethnopharmacology, while technical advances in metabolomics and biochemometrics have revolutionized the field. These insights are the opinions and reviews of the authors only. They are directed to early career scientists in the field or pharmacologists without phytochemical training rather than directed to experienced phytochemists. The insights are intended to help them better understand modern phytochemical requirements in publications as well as advances in phytochemical methods which improve quality of ethnopharmacology studies.

## Methods

This article is not a comprehensive review and only a few representative examples of studies were selected from articles found using the databases, PubMed or Web of Science (Clarivate™) from January 2005-October 2021 using keywords including phytochemical methods, targeted and untargeted analysis, chemometrics, biochemometrics, phytochemical metabolomics, active principle identification, and dereplication. Technical details are limited, and readers are directed to the referenced papers for detailed information about equipment, proprietary software and statistical packages, etc. Two major areas covered are (1) pharmacopeial plant preparations and commercial medicinal plants and (2) new phytochemical studies of traditional plant medicines and plant drug discovery. While these areas could apply to medicinal plants from any part of the world, our examples come from the Americas and provide current examples from here.

## Pharmacopeial Plant Preparations and Commercial Medicinal Plants

For defined pharmacopeial plant preparations and commercial medicinal plants, a primary objective is to characterize phytochemicals for identity and quality assurance.

### Identity Confirmation, Substitution and Adulteration in Commercial Medicinal Plants

**FIGURE 1:**
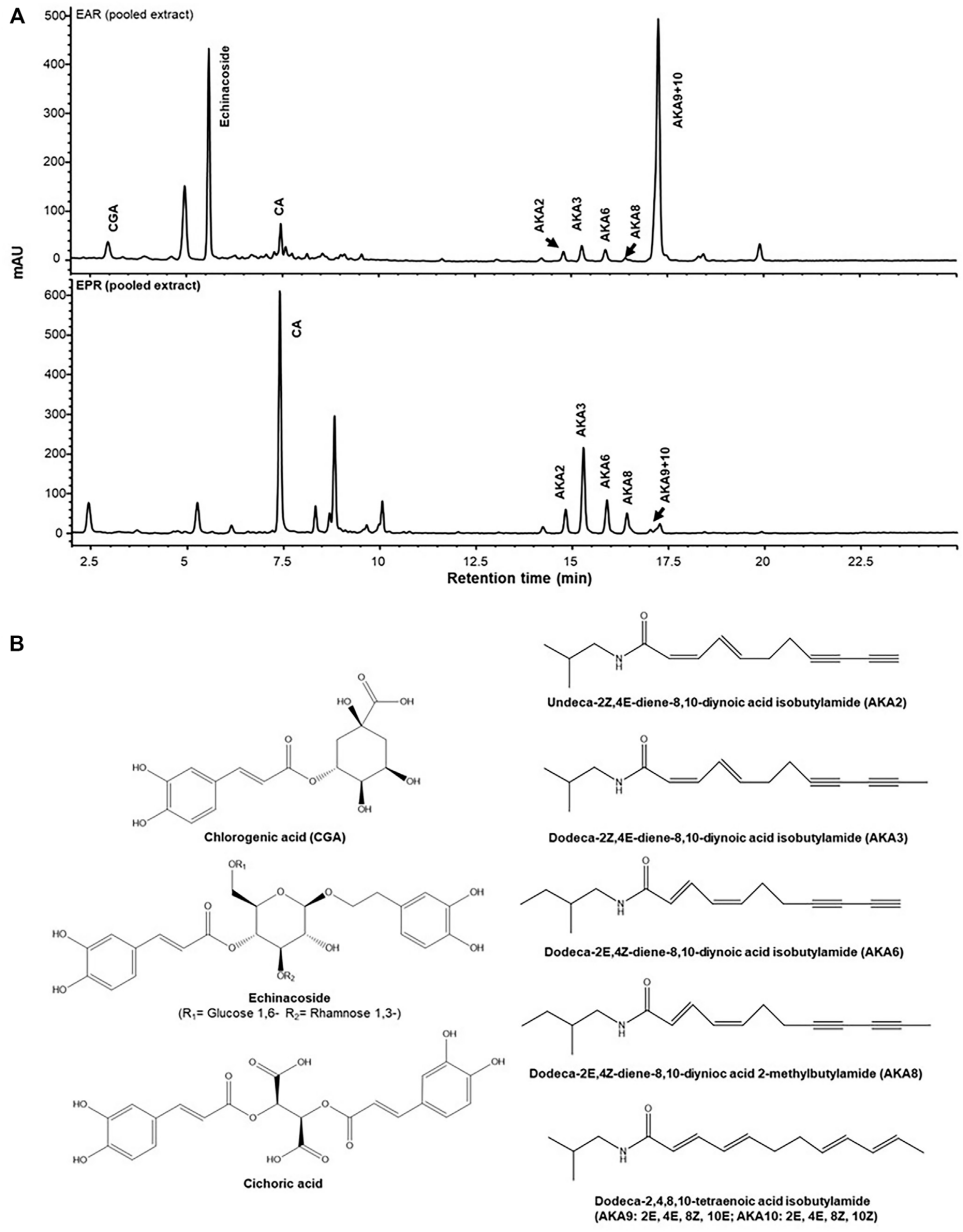
Targeted identity analysis of *E. purpurea* (EPR) and *E angustifolia* (EAR) roots by HPLC-DAD-MS showing distinct phytochemical profiles for each species **(A)**. Quantification of these markers, **(B)** with a validated method provides quality assurance for pharmacological studies. Reproduced from Liu R., Salas N.C., Li W., Wang L., Arnason J.T., Harris C.S. Interactions of *Echinacea* spp. root extracts and alkylamides with the endocannabinoid system and peripheral inflammatory pain. *Frontiers in pharmacology.* 2021 12:776. Reproduced under the terms of a CC BY 4.0 license https://creativecommons.org/licenses/by/4.0/.

Identity can be difficult to establish since raw plant products are often received by finished product formulators as internationally shipped dried powders from independent supply houses. Adulteration and substitution of species are common issues. Since plant morphological feature are absent, industry and regulators have traditionally confirmed identity by phytochemical analysis targeting one or a few species chemical markers. In industry, identity is still commonly achieved by liquid chromatography systems coupled to diode array and ultraviolet-light detector (LC-DAD, LC-UV), or mass spectrometry (LC-MS) detector using co-analysis with isolated standards of the marker compounds purified to >95%. For example alkylamides identified originally by Bauer et al. (1988) as well as well-known caffeic acid derivatives are typically used in pharmacopeial monographs as phytochemical evidence of identity of *Echinacea purpurea* L. Moench (Asteraceae) (Upton 2004). Examples of chromatograms used for distinguishing *E. purpurea* and *E. angustifolia* DC are shown in Figure 1 (Liu et al., 2021a). High performance thin layer chromatography (HPTLC) methods are also accepted (Upton 2004; Gafner et al., 2021) but older colorimetric tests such as total content assays of phenolics, tannins, saponins, flavonoids, alkaloids are no longer accepted in this journal or by most regulators.

Identity is very important because substitutions and adulterations are often encountered that can be dangerous to consumers. A well-known case of substitution involved North American Black Cohosh [*Actea racemosa* L. (Ranunculaceae)] products used for symptoms associated with menopause. Mahady et al. (2008) identified thirty unduplicated reports concerning liver damage with the use of black cohosh products. All the reports of liver damage were assigned possible causality, and none were probable or certain causality. One high profile case of liver damage in Canada received analysis with respect to authenticity of the product because there were few confounding factors (the patient had no underlying conditions, was taking no other drugs and consumed just one identified commercial product sold as black cohosh). Identity and registration of the commercial product was originally established by the presence of two markers, actein and 23-epi-26-deoxyactein. However, these compounds are also found in several other Asian species in the genus. More rigorous analysis of the product for several chemical markers led to the discovery of the presence of cimifugin, an antipruritic compound, present in an Asian species but not black cohosh from North America. Because of this case, the analytical requirements for registration in Canada have been updated to include cimiracemide C as an *A. racemosa* marker and to exclude materials containing cimifugin and other Asian species markers to ensure the plant material has not been substituted or adulterated (Health Canda, 2018).

There have been several recent incidents of adulterations in North America due to herbal supply issues in the Covid 19 pandemic. One example is great interest in antiviral botanical extracts derived from elderberry [*Sambucus nigra* L. and *S. canadensis* L. (Viburnaceae)] which saw great demand and short supply, leading to appearance of adulterated products (Gafner et al., 2021). Industry -independent groups such as the American Botanical Council’s Botanical Adulterants Prevention Program (PAP) published in the trade magazine HerbalGram (https://www.herbalgram.org) established that adulterated products were common in elderberry products marketed in North America (58 products out of 532 analysed by collaboration independent labs by HPLC-DAD or UV-Vis, or HPTLC) and often contained black rice extract which contains the elderberry marker cyanidin-3-*O*-glucoside. The presence of peonidine-3-*O*-glucoside and other markers are indicative of black rice extract. Therefore robust analytical methods are needed to prevent substitution and identity issues in a wide variety of problem commercial products such as methods provided by United States Pharmacopeia (USP).

### Metabolomics and Chemometrics

Untargeted metabolomics and chemometrics have recently accelerated the authentication of identity and identification of adulterants by high-throughput profiling (Ravaglia et al., 2019; Wallace et al., 2020). Metabolomic methods are becoming more accessible and overcome many of the limitations associated with genomic- and simple biomarker-based techniques (Simmler et al., 2018).

The availability of metabolomics platforms and methods allow rapid annotation of hundreds of small molecules by the comparison of their spectrometric features (m/z values and retention times) in spectral databases. This provides a technicolour view of plant phytochemistry where we once saw only the black and white version of extracts. In the majority of studies, the metabolome is identified by LC-MS or LC-MS/MS methods, where compounds in the injected extract are first separated by HPLC or UPLC systems and then detected either using a time of flight (TOF) or Orbitrap MS detector. Compounds are annotated from their mass spectrometry m/z ion and chromatography retention time data. They can be identified with different levels of accuracy using in house and online data deposited in spectral libraries. This analysis attempts to identify any small molecule present, it is therefore called untargeted. Each identified compound or annotated m/z signal is a chemotaxonomic character and a large number of them greatly improves identification of species, or difficult to separate varieties and different type of extracts.

Chemometrics (Statistical methods are applied to chemical analysis) can classify plant extracts into different taxa (or other categories such as type of extract) through uni or multivariate statistical methods like principal components analysis (PCA). Once a series of known extracts is analysed and classified, an unknown can be analysed and classified based on its metabolome grouping (or exclusion). Further analysis such as orthogonal partial least square discriminant analysis (OPLS-DA) or hierarchical clustering (heat maps) are very powerful statistical methods which can identify significantly different phytochemical markers in contrasted extracts subjected to metabolomic analysis.

Brown et al. (2012a and b) applied UPLC-MS-TOF chemometrics to identity three species of cranberry and in a second study five cultivars of commercial North American cranberry cultivars. Partial least squares discriminant analysis (PLS-DA) was applied to the metabolomics data and led to the separation of each species. In the cultivar study, most signals were detected in all extracts but a few were identified as associated with specific cultivars. Whereas PCA of the cultivar phytochemistry did not lead to the full separation of cultivars, PLS-DA exhibited significant clustering of extracts by cultivar, indicating that the method can separate and identify cultivars. Recently Mudge et al. (2019) have used chemometric methods to study cannabinoid and terpene chemotaxonomy in cannabis.

**FIGURE 2:**
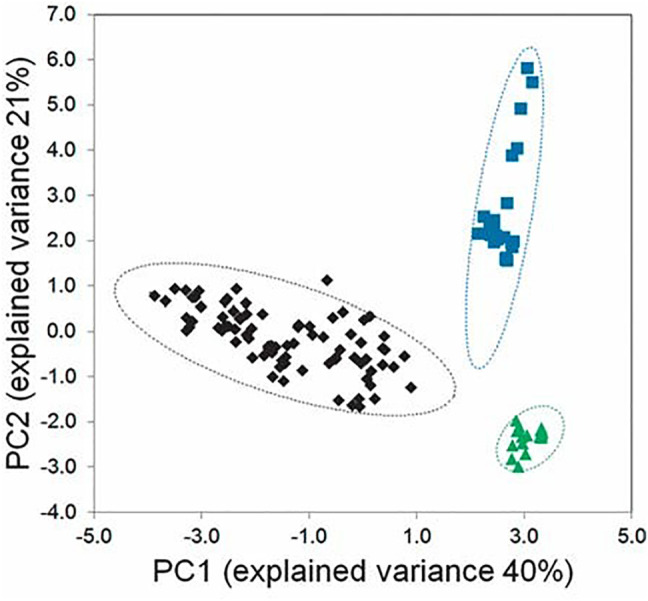
Chemometric analysis of Vaccinium species using NMR metabolomic data. Scores plot for three species, *Vaccinium angustifolium* (black diamonds), *V. ovalifolium* (blue squares), and *V. macrocarpon* (green triangles). Hotelling ellipses, showing the region of the 95% confidence limit, are shown for each group by dashed lines of the matching color. Note that spectra for each species occupy a distinct region, with no overlap. Reproduced (Figure 2) with permission from Markus, M. A., Ferrier, J., Luchsinger, S. M., Yuk, J., Cuerrier, A., Balick, M. J., & Colson, K. L (2014). Distinguishing Vaccinium species by chemical fingerprinting based on NMR spectra, validated with spectra collected in different laboratories. Planta medica, 80(08/09), 732-739.

An alternative method of chemical fingerprinting is based on high-resolution nuclear magnetic resonance (NMR) spectroscopy. For example, Markus et al. (2014) studied NMR spectra of several *Vaccinium* spp. (Ericaceae). Instead of identifying specific compounds, the NMR spectra from submitted samples were binned (divided into many different subspectra) that were used as taxonomic characters for statistical analysis. Using PCA, leaf extracts of the three species were resolved in the scores plot (Figure 2) and analysis of variance showed that the three species differed significantly, establishing that the species can be distinguished by NMR alone. Because NMR spectra do not vary greatly from instrument to instrument when magnet size is basically the same (i.e. 500 or 600 MHz), and this equipment is available at many institutions, the method was validated with spectra collected in six different institutions. Using blinded samples from the reference set*, V. ovalifolium Sm.* and *V. macrocarpon* Aiton were classified correctly 100% of the time and *V. angustifolium* Aiton 94% of the time. These NMR methods have high resolving power and have already been adapted for a large number of blueberry species (Ferrier, 2014) and for industrial use in the rapid identity control of fruit juices of different geographic origin.

Plant powders can also be identified by near infrared (IR) reflectance spectroscopy, since this region of the electromagnetic spectrum also provides good structural information (Türker-Kaya and Huck., 2017). This method is inexpensive and generally has good reliability for identification of different common medicinal plants.

Comparing methods, neither the NMR metabolomic nor IR methods provide the rapid identification of marker compounds afforded by LC/GC-MS methods. NMR metabolomics is somewhat limited by low sensitivity and (unresolvable) overlapping signals compared to LC-MS. LC-MS methods, specifically those with high resolution analyzers, rapidly lead to separation and identification of known compounds. Major obstacles, however, include the expensive purchase and operating costs, the need for experienced operators, and the lack of robust spectral libraries to support accurate chemical identification. Nonetheless, researchers and young scientists with early access to this equipment enthusiastically attempted to identify many new compound occurrences in plants with LC-MS techniques. Because of positional ambiguity in MS identification, a former editor of Phytochemistry (journal) was concerned that the early metabolomic papers contributed many tentative structural assignments to the published scientific literature that were incorrect. Improved procedures in the last decade and use of MS/MS and TOF data (accurate mass values) with more rigorous online spectral libraries have made identification of phytochemicals much more reliable. On the other hand, early untargeted metabolomic studies led to important new discoveries such as the identification of the animal hormone melatonin in plants, which has led to a new field in plant physiology (Murch et al., 1997).

### Quality Requires Quantitative Validation Studies

While profiling of metabolites for identity may be sufficient for early mechanistic studies of plant drugs, advanced ethnopharmacological studies in animal models of clinical settings cannot be compared or repeated without quantitative assessment of the main active principle(s). There is no shortcut to quantitation which requires prior quantitative validation studies that use highly purified phytochemical standards and strict adherence to International Harmonization Standard (IHS) validation methods, usually by LC-DAD-UV or LC-MS methods. These include characterizing the method in terms of precision, accuracy, linearity, interlab and intra-/inter-day variability, Limit Of Detection, Limit Of Quantification, etc. Recovery of standards from spiked samples are recommended to determine how accurately the method captures and quantifies targeted compounds. A recent example (Mamallapalli and Raju, 2021) is a validated method for kava (*Piper methisticum* G. Forst. (Piperaceae)), in which they developed a UPLC MS/MS-based analytical method for the expanded quantification of six major kavalactones (kavain, dihydrokavain, methysticin, dihydromethysticin, yangonin and desmethoxyyangonin) and two new flavokavains. They used labelled isotope standards which improved recovery estimates. Many validated methods for pharmacopeial products are published or available from USP and the Association of Official Agricultural Chemists (AOAC).

### Plant Metabolomics Meets Network Pharmacology

Network pharmacology is a systems approach to pharmacology that combines systems biology, network analysis and bioinformatics, and it is characterized as “the next paradigm” in drug discovery (Hopkins, 2008). Originally it was conceived as a study of single entity drugs activating pharmacological receptor(s) leading to a network of interacting downstream cellular events. The array of secondary metabolites identified in metabolomic studies of medicinal plants is now acknowledged to activate several signaling pathways that can lead to multiple independent and synergistic or interacting effects. This field has recently been explored in detail and is found in the special issue of Frontiers in Pharmacology: ethnopharmacology, entitled Network Pharmacology and Traditional Medicine (Lai et al., 2020). In this issue, many interesting examples of the application of network pharmacology are found in the complex formulae of Traditional Chinese medicines which provides a new strategy and powerful tool to uncover the biological basis underlying herbal formula.

## New Phytochemical Studies of Traditional Plant Medicines and Plant Drug Discovery

At the turn of the millennium, major research groups were funded by US drug discovery grants for work on traditional indigenous medicines in North and South America. These programs combined drug companies, researchers and Indigenous people to find new single entity drugs for development from traditional medicines. Some of these programs were controversial and criticized by Indigenous groups as commercial appropriation of Indigenous knowledge. In response, ethical guidelines were developed internationally which led to the Nagoya protocol, an amendment to the Convention on Biological Diversity (co-chaired by Canada in 2014) which provided a framework for access to biodiversity and benefit sharing with Indigenous people. Unfortunately, the co-chair country, Canada, did not consult with Canadian Indigenous First Nations in Canada on a timely basis and did not receive their approval of the treaty. The protocol has been rejected by a consortium of First Nations (Grand Council of the Crees, 2012) as both a colonial process and document that violates their sovereignty and treaty rights. Fortunately, efforts to reset the relationship with Indigenous people are underway, in which the voices and leadership of First Nations in research are being heard (Styawat/Joseph, 2022).

As explained to our team by Cree and Maya indigenous healers with whom we have worked closely for decades, they do not wish to commercialize their plants, as they believe the healing properties of plants are a sacred gift of the creator. The priority of healers is to personally treat patients and not the development of commercial medicines or drugs. However, they value modern scientific studies, when they can provide information on the safety and efficacy of traditional medicines. They believe that this scientific translation of traditional knowledge is key to recognition and respect from government regulators and others. To achieve this goal, the identification of active principles and assessment of pharmacological effects (efficacy and safety) is appropriate.

Alternatively, some Indigenous groups including a few in Canada have accepted commercialization, if the process can be done sustainably and fairly. In the field of plant drug discovery many groups internationally believe that if the Nagoya protocols are followed and benefit sharing agreements are in place with Indigenous groups, this represents current best practice. Efforts to improve trust and partnership for research on traditional medicines are now being developed, for example in Guatemala (Berger-Gonzalez et al., 2022). In this case, plant-based traditional medicines may be studied to find new active principles, which may lead to standardized and tested medicinal plant drugs or become active lead compounds for chemical analoging to improve efficacy, availability and safety useful for the treatment of well-known diseases as well as emergent and culture-bound conditions.

### Classic Bioassay Guided Isolation

**FIGURE 3:**
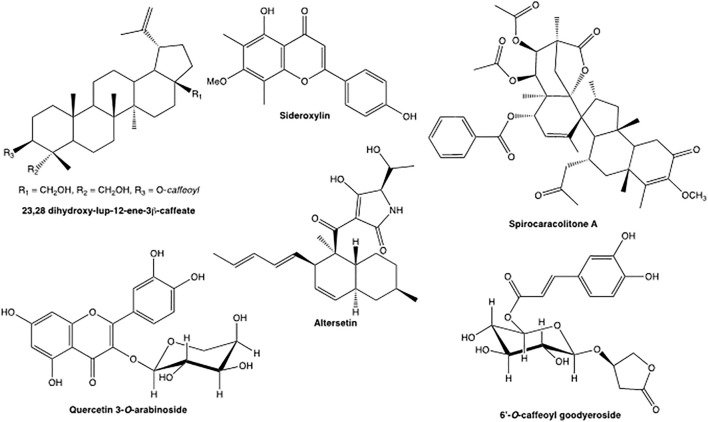
New bioactive compounds from plants and fungi.

The classic method to determine active principles in medicinal plants is bioactivity guided isolation (BGI). An example of classic BGI for ethnopharmacology is the isolation of antidiabetic compounds from Cree traditional medicines. Showy mountain ash bark (*Sorbus decora* (Sarg.) C.K. Scneid. (Rosaceae) is used by traditional Eyou Istchee Cree healers in the James Bay district of Northern Quebec for symptoms of diabetes. The extracts of bark were studied in a glucose uptake assay with muscle cells and found to have activity that compared favorably with metformin. The plant extract was successively fractionated by column chromatography and fractions tested to find activity (Guerrero-Analco et al., 2010), before a new round of purification was undertaken, and eventually pure compounds isolated by preparative HPLC and identified by spectroscopic methods. A new triterpenoid, 23,28-dihydroxylupan-20(29)-ene-3β-caffeate was identified with exceptional activity for stimulation of glucose uptake in muscle cells (Figure 3).

**FIGURE 4:**
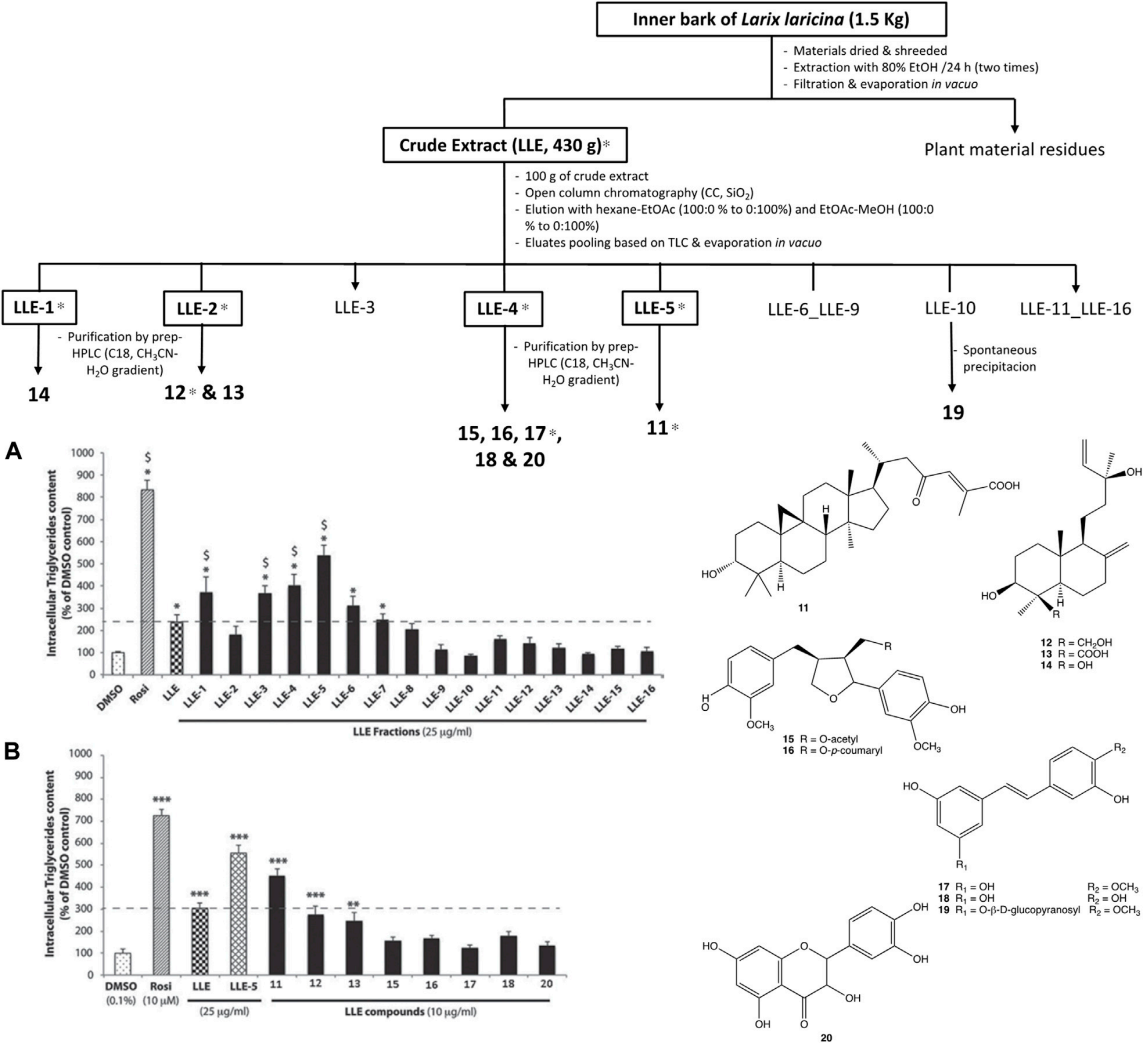
Classic bioassay guided fractionation of *Larix larcina* (Tamarack or larch) in an adipogenic activity assay leading to identification of a novel cycloartane triterpenoid named Awashishic acid (23-oxo-3 -hydroxycycloart-24-en-26-oic acid, 11). Figure adapted with permission to use data from Shang et al., 2012. Adipogenic constituents from the bark of Larix laricina du roi (K. koch; Pinaceae), an important medicinal plant used traditionally by the Cree of eeyou istchee (Quebec, Canada) for the treatment of type 2 diabetes symptoms. J. Ethnopharmacol.141:1051-7.

In a related study (Shang et al., 2012), the adipogenic active principles of the bark of Tamarack trees, *Larix larcina* (Du Roi) K. Koch. (Pinaceae) were isolated by a similar approach (BGI). The bark is used for the treatment of diabetic sores, frequent urination, and infected wounds by Cree healers. It was found to significantly enhance adipogenesis in differentiating 3T3-L1 adipocytes in a screening study using *in vitro* cell-based bioassays. *L. laricina* extract decreases hyperglycemia and insulin resistance *in vivo*, using the diet induced obese mouse model. The cell line was used to carry out a BGF of an 80% EtOH extract from the inner bark of Tamarack. A total of 16 primary fractions were separated on a normal phase silica gel column (SiO_2_) and the most active one had two thirds of the activity of the reference drug rosiglitazone. Ten compounds were further isolated by preparative HPLC and the most active one was a novel cycloartane triterpenoid, 23-oxo-3α-hydroxycycloart-24-en-26-oic acid, which strongly potentiates adipogenesis with an EC_50_ of 7.7 µM (Figure 4). This compound was name awashishic acid to honour the contributions of a Cree elder and healer, Sam Awashish.

The action of medicinal plants is often attributed to multiple active principles and interacting synergists. Junio et al. (2011) developed a bioassay guided procedure to identify synergists of the antimicrobial alkaloids in golden seal (*Hydrastis canadensis* L. (Ranunculaceae)). Golden seal was fractionated by column chromatography and the fractions were tested in a synergy assay for antimicrobial activity against *S. aureus*. The assay combined the active principle berberine with the fraction tested over a concentration range of 5–300 μg/ml in a broth dilution assay. After two rounds of fractionation the synergist compounds were isolated by preparative HPLC and identified by ^1^H- and ^13^C-NMR as the flavonoid sideroxylin (Figure 3) and its derivatives. Its mode of action was found to be the inhibition of the alkaloid efflux pump in the pathogenic bacteria.

### Newer Methods in Identifying Bioactivity Markers

The disadvantage of classical BGF has been the need for large amounts of plant material (200–1500 g) and massive gravity columns to separate enough pure phytochemicals (2–3 mg) for structure elucidation by 1- and 2D-NMR spectroscopy and biotesting. A project like this often takes several months. Recent advances in technology now enable this process to be scaled down to 20 mg plant extract with rapid results achieved by integrating small scale bioassays with zebrafish. Bohni et al. (2013) developed a microscale bioassay guided platform for the study of the anti-angiogenic activity of a traditional medicine, *Rhynchosia viscosa* (Roth) DC (Fabaceae). They used rapid microfractionation of extracts, and microflow NMR methods allowing the identification of µg amounts of pure phytochemicals. They successfully screened these in zebrafish anti-angiogenic assays. The anti-angiogenic compounds identified were genistein and sophora isoflavone. Active principle identification can now be achieved rapidly using this and other microscale platforms.

Biochemometric analysis is an alternative method for the identification of bioactive compounds combining metabolomics, bioactivity studies and statistics and greatly reducing the need for assaying many column fractions. Nowadays, combination of bioassay and metabolomics guided isolation/fractionation are commonly used.

We undertook application of biochemometric analysis in a study of Echinacea phytochemicals active at cannabinoid targets relevant to a peripheral pain model (Liu et al., 2021a; Liu et al., 2021b). With a large number (40) of variable individual plant accessions of *Echinacea angustifolia* and *E. purpurea* available grown in a similar environment, linear regression of activity against concentration of phytochemical was feasible. Analyses of extracts were undertaken by HPLC DAD-MS for caffeic acid derivatives and alkamides and they were assayed for or CB1 and CB2 agonist activity or inhibition of fatty acid amide hydrolase (FAAH). Some of the results obtained with linear regression revealed CB2 agonist activity was positively and significantly (*r* ^2^ = 0.43 and *p* = 0.03) related with the major isomeric alkylamides, dodeca-2E, 4E, 8 Z, 10 E/Z tetraenoic acid isobutylamide in *E. angustifolia.* Caftaric acid, cichoric acid and one isobutyl amide are the strongest determinants of activity for FAAH inhibition in *E. pupurea* root. Results were confirmed with isolated compounds in the bioassays and in a rat model of inflammatory peripheral pain using a CB2 antagonist.

**FIGURE 5:**
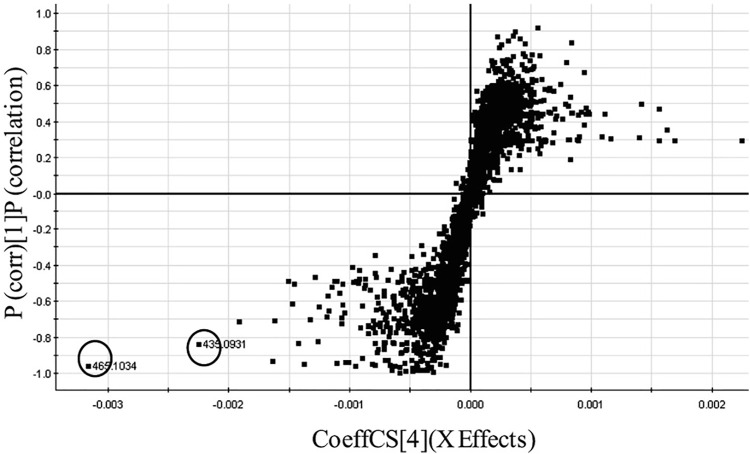
S-plot of active versus inactive Cree plants leading of two significant biomarkers (circled) of inhibition of glucose uptake, quercetin 3-O-arabinoside and quercetin 3-O-galactoside. Figure appears as inset of Figure originally published in Shang et al. (2015). Novel approach to identify potential bioactive plant metabolites: pharmacological and metabolomics analyses of ethanol and hot water extracts of several Canadian medicinal plants of the Cree of Eeyou Istchee, PLoS One, 2015, 10(8), e0135721. Reproduced from Ref. 19, under the terms of a CC BY 4.0 license https://creativecommons.org/licenses/by/4.0/

In another experiment, we used discriminant analysis to compare 17 Cree medicinal plant extracts that were very active in a antidiabetic assay for glucose uptake in C2C12 muscle cells, with species that displayed low activity (Shang et al., 2015). The metabolome of each extract was recorded using UPLC coupled to a quadrupole time of flight (QTOF)-MS. In this hyphenated technique, complex extracts were separated into 400+ compounds through a small-bore reverse-phase column at very high pressure. Both the PCA and OPLSD plot showed separation of the high activity and low activity medicinal plants. Using an S-Plot analysis of the data to contrast the high activity and low activity species (Figure 5), two significant biomarkers for activity were discovered in the active plants and identified as quercetin-3-*O*-galactoside and quercetin 3-*O*-arabinoside (Figure 3) based on their accurate MS spectra. The compounds were tested in the antidiabetic assay and bioactivity was confirmed.

Discriminant methods can also be used with the fractions of one bioactive species (Kellogg et al., 2016), and comparisons of several methods were made to find the best statistical procedure. Fractions of the endophyte fungi *Alternaria* sp.(Deuteromycetes) and *Pyrenochaeta* sp. (Ascomycetes) were assayed for antimicrobial activity against the bacteria *Staphylococcus aureus* and analysed by LC-MS/MS. In the biochemometric analysis, significant antibacterial ions could be identified in fungal extracts using the S-plot analysis described above. However, they also evaluated a different statistic called the selectivity ratio which represents a quantitative measure of each variable’s power to distinguish between different groups, and found it to be the best parameter for identifying bioactive ions from these extracts. In the study the method identified the antibacterial compounds altersetin (MIC 0.23 μg/ml) (Figure 3) and macrosphelide A (MIC 75 μg/ml).

This biochemometric approach using the selectivity ratio was also employed in a recent study of fractions of curled dock, *Rumex crispus* L. (Ranuculaceae)*,* an indigenous remedy for diarrhea and skin infections studied here for antimicrobial activity (Pelzer et al., 2021). The selectivity ratio evaluated for fractions led to the identification of ten active antibacterial compounds including anthraquinones such as emodin and several of its derivatives, *iso*-ferulic acid, and scopoletin.

The biometric approach is also applicable to identifying synergists as revisited in goldenseal by Britton et al. (2017).

### Dereplication

A challenge in ethnopharmacology is that it is important to find new active molecules in early stages of the chemical characterization or isolation, which can be achieved by a process known as dereplication. Conventionally this has been achieved in analytical methods combined with data bases of known compounds for comparison. Extracts with unidentified peaks are prioritized for isolation for pure compounds which are identified by conventional spectroscopy (1 and 2D NMR, IR and high-resolution MS). For example, El-Elimat et al. (2013) developed a method for dereplication of fungal metabolites. UPLC-PDA-HRMS-MS/MS was used for analysis of secondary metabolites in crude culture extracts and a database of 170 known fungal metabolites was constructed by recording HRMS and MS/MS spectra of fungal metabolites, utilizing both positive- and negative electrospray ionization modes. Cultures of 106 fungi were grown on a small scale extracted and tested for cytotoxicity to human cancer cell lines in culture, including MCF-7, H-460, and SF-268 cells. Active extracts were then analysed and only 55 containing unidentified peaks were prioritized for further isolation work.

With chemometrics and network pharmacology combined, powerful new methods are now available. An approach adopted for identification of antiviral compounds from *Euphorbia dendroides* L. (Euphorbiaceae) was developed by Nothias et al. (2018). After fractionation, each fraction was assessed by LC-MS and bioactivity scores were made using the relative abundance of a molecule in fractions and the bioactivity level of each fraction. Next, they assessed the potential bioactivity. First, they used a targeted approach, where prior knowledge was used, such as chemotaxonomic information and the sets of previously described bioactive molecules. Second, they used an untargeted approach by looking for consistent patterns of bioactive candidates in networks, such as clusters of phytochemicals with a high frequency of bioactive candidates. Cluster V in their study was identified as a priority and several deoxyphorbol ester derivatives were isolated by preparative HPLC in the study. Evaluation of the biological activity indicated two compounds that are selective inhibitors of Chikungunya virus replication in the submicromolar range.

An alternative approach to chemical dereplication is biological dereplication. It is well known that the distribution of bioactive phytochemicals in plants is specific to particular plant families. As a consequence, we undertook a phytochemical discovery program based on uninvestigated rare families are likely to be a source of novel phytochemicals. We applied this method to plants in the Sarracineaceae (Pitcher plant family) and the Lepidobotryaceae (tropical family with two genera, with few species)*. Sarracinea purpurea* L. Moench (Sarracineaceae) , an antidiabetic plant used by Cree healers yielded over 13 phytochemicals including the novel 6′-O-caffeoyl goodyearoside (Figure 3) which was a new glucose uptake inhibitors (Muhammud et al., 2013). *Ruptiliocarpon caracolito* Hammel & N. Zamora (Lepidobotryaceae) (Asim et al., 2010) a plant used as a snuff by shaman in South America, yielded 17 novel spiro triterpenoids including spirocaracolitone A (Figure 3) with insecticidal and anxiolytic activity.

Recently, Wolfender’s group has provided a review and synthesis of dereplication methods combining Liquid Chromatography–High Resolution Tandem Mass Spectrometry, NMR profiling, *In silico* studies, Spectral Databases, and Chemometrics analysis (Wolfender et al. (2018); Allard et al., 2016).

## Conclusion

Consideration of some of the issues in this review by researchers can help them avoid defficiences leading to rejection of publications as well as lead to positive relationships and outcomes with First Peoples. The selected studies described in this review illustrate in part the rapid advances in technology and knowledge that is occurring in Phytochemistry and Ethnopharmacology today. The discipline has clearly moved from qualitative descriptive studies to quantitative, statistically rigorous science. There are many uninvestigated plants to work on and we are truly at the beginning of a great age of drug discovery and scientific understanding of traditional knowledge.
